# Behavioral Variant Frontotemporal Lobar Degeneration with Amyotrophic Lateral Sclerosis with a Chromosome 9p21 Hexanucleotide Repeat

**DOI:** 10.3389/fneur.2012.00136

**Published:** 2012-10-04

**Authors:** Robert P. Friedland, Jignesh J. Shah, Lindsay A. Farrer, Badri Vardarajan, Jovan D. Rebolledo-Mendez, Kin Mok, John Hardy

**Affiliations:** ^1^School of Medicine, University of LouisvilleLouisville, KY, USA; ^2^Boston University School of MedicineBoston, MA, USA; ^3^Rita Lila Weston Research Weston Laboratories, Department of Molecular Neuroscience, University College LondonLondon, UK

**Keywords:** dementia, frontotemporal lobar degeneration, amyotrophic lateral sclerosis, motor neuron disease, ubiquitin, disinhibition, hexanucleotide repeat

## Abstract

To determine the genetic basis of familial frontotemporal lobar degeneration (FTLD) with amyotrophic lateral sclerosis (ALS) we performed a clinical and genetic analysis of an affected family. A 51-year-old man with behavioral variant FTLD with ALS had a family history of the disease suggestive of autosomal dominant inheritance with incomplete penetrance. Genetic studies in this patient demonstrated the presence of an amplified hexanucleotide repeat (>30) polymorphism in the chromosome 9 open reading frame 72 (*C9ORF72*) gene which was previously identified as a cause of FTLD. Five others unaffected from the family were negative (all had less than 11 repeats). Because of the clinical and pathological overlap between FTLD and AD we performed a larger genome-wide association study and did not find association of single nucleotide polymorphisms (SNPs) in the *C9ORF72* gene with Alzheimer’s disease (AD) risk. Bioinformatic analysis of *C9ORF72* using the Gene Expression Omnibus database showed expression differences in patients with muscular dystrophy, neural tube defects, and schizophrenia. We also report analysis of gene expression in brain regions using the Allen Human Brain Atlas. Defects in this recently reported gene are now believed to be the most common cause of inherited ALS and an important cause of inherited FTLD. Our work suggests that the gene may also be important in other neurological and psychiatric conditions.

## Introduction and Background

Frontotemporal lobar degeneration (FTLD), originally described by Alois Alzheimer’s contemporary Arnold Pick, has been recognized as the second leading cause of dementia in people under the age of 65 (Ratnavalli et al., 2002). Its behavioral variant causes abnormal social behavior characterized as disinhibition with emotional lability and inappropriate laughter. FTLD may be found alone or accompanied by corticobasal degeneration, parkinsonism, or amyotrophic lateral sclerosis (ALS). As many as 50% of ALS patients have cognitive and behavioral deficits and about half of FTLD cases have motor neuron impairment. One quarter to one half of FTLD cases have an autosomal dominant pattern of inheritance and several genes on chromosome 9p have been linked to familial FTLD with ubiquitin immunoreactive inclusions (Vance et al., 2006; Liscic et al., 2008). A genetic locus associated with FTLD with ALS has recently been identified as a hexanucleotide repeat located on chromosome 9, open reading frame 72 (*C90RF72*; Mok et al., 2012; DeJesus-Hernandez et al., 2011; Laaksovirta et al., 2010). This gene is believed to be the most common cause of familial FTD-ALS. We describe an additional case and report results of a genetic association analysis in a very large Alzheimer disease (AD) dataset and a bioinformatic analysis demonstrating altered expression of *C9ORF72* in patients with other neurological conditions and expression patterns in the brain.

The studies described below were approved by the Institutional Review Board of the University of Louisville Health Sciences Center and informed consent was obtained from all subjects.

## Case Report

A 51-year old Caucasian man presented to the hospital with falls beginning in October, 2010. Weakness and retraction of fingers in both hands with gradual cognitive decline were noticed at that time and he could no longer take care of himself, needing help with bathing, with wandering, denial of illness, and inappropriate emotional expression. When he was asked why he was in the Emergency Room, he said that his computer did not work. Past history, social history, and review of systems were not contributory. Mental status exam demonstrated moderate dementia with word finding difficulties, trouble naming object parts, inappropriate laughter, motor impersistence, inability to follow commands, impaired calculations, inappropriate smiling, and laughing throughout the exam. There was diffuse weakness and wrist drop bilaterally with muscle atrophy in the hands and legs, difficulty walking, and fasciculations in all four extremities. Sensory exam and deep tendon reflexes were normal. Laboratory tests for routine studies and non-genetic causes of ALS or dementia were negative. Positron emission tomography with 18-fluorodeoxyglucose was compatible with advanced frontotemporal dementia with bilateral anterior frontal and anterior temporal hypometabolism, including the cingulate gyrus. Glucose metabolism was relatively preserved in the basal ganglia. MRI imaging of the brain showed diffuse cortical atrophy.

## Family History

**Figure 1:**
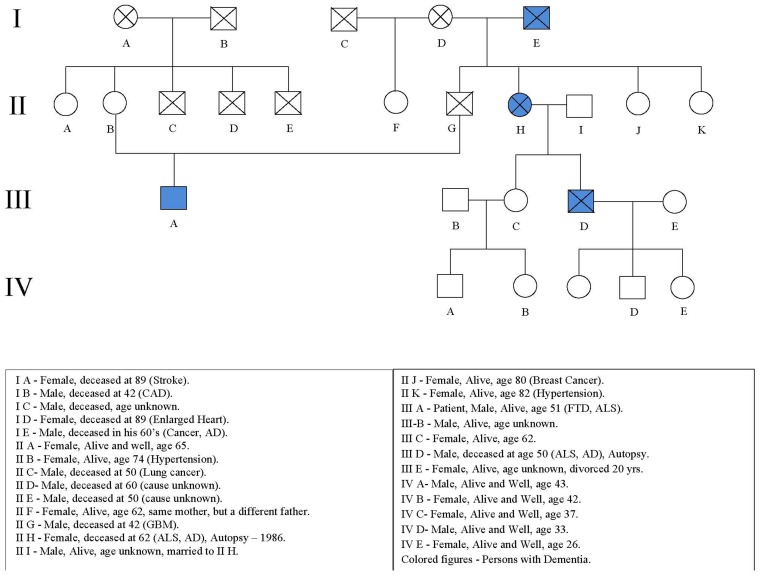
**Pedigree**. The proband is subject IIIA.

The patient’s mother is alive and well at age 74. His father died of a brain tumor at age 42. His father’s sister had early onset dementia at age of 50 with ALS and died at age 62 (IIH; Figure 1). Her son also had early onset dementia with ALS and died at the age of 59 (IIID). The patient’s paternal grandfather also had dementia and died in his 60’s (IE).

An autopsy performed in 1991 of the patient’s father’s sister (IIH) demonstrated severe cerebral cortical atrophy, most prominent in the frontal poles, and to a lesser extent in the parietal and temporal lobes. The cerebellum was also atrophic. The lateral ventricles were symmetrically enlarged as was the third ventricle. The ventral roots were small and demyelination of the lateral columns was seen in the cervical enlargement. Degenerative changes in nerve cells were observed in the cortex without gliosis. Senile plaques or neurofibrillary tangles were not detected. Loss and degenerative changes of the anterior horn cells was observed with thinning of the anterior roots.

The patient’s first cousin (IIID; Figure 1) died in 2009 at age 59 with dementia, fasciculations, weakness, spasticity, hypereflexia, and cortical atrophy after an illness of 1 year duration. Autopsy demonstrated atrophy of the spinal cord with neuronal loss and gliosis in the anterior horns, cortex, hippocampus (CA1 and subiculum), hypoglossal nucleus, and basal ganglia. Ubiquitin positive inclusions were found in cell bodies and neurites in the right and left hippocampi including the granular cells of the dentate gyrus, the tegmentum of the medulla, and the gray matter of the spinal cord. Neurofibrillary tangles were found in the neurons in the right and left hippocampi. Amyloid Beta protein deposition was not detected.

Incomplete penetrance is suggested by the absence of the disease in four out of the five children of affected subject IE (the proband’s paternal grandfather) and also the absence of disease in three out of the four children of affected subject IIH (the proband’s father’s sister). The pedigree was altered to preserve confidentiality.

## Results: Genetic Testing

The proband was positive for a non-coding GGGGCC hexanucleotide repeat in the gene *C9ORF72* (>30 repeats). Five other unaffected from the family were negative. All had less than 11 repeats.

### Association of *C9ORF72* with alzheimer disease

We tested the association of 45 genotyped and imputed single nucleotide polymorphisms (SNPs) in *C9ORF72* with risk of AD in a large genome-wide association study (GWAS) dataset containing 8,309 late onset AD cases and 7,366 controls (Naj et al., 2011). Nominally significant results were observed with four SNPs (*p* < 0.05), but none of these findings remained significant after correction for multiple testing. Association with *C9ORF72* was also not significant (*p* = 0.12) using the VEGAS gene-based test method (Liu et al., 2010). These findings suggest that variants in *C9ORF72* do not influence AD risk.

### Bioinformatic analysis of *C9ORF72*

Because the function of *C9ORF72* is unknown, we queried the Gene Expression Omnibus (GEO) database at the National Center for Biotechnology Information (NCBI), one of the largest public repositories for gene expression data^1^, for evidence of differential expression of this gene in brain or the CNS. This database stores individual gene expression profiles from curated “DataSets” and can be used to search for profiles of interest based on gene annotation or pre-computed profile characteristics.

Three independent searches were performed on the GEO profiles database. These searches were based on the “*Flag Type*” field in the GEO profiles database which categorizes profiles that exhibit specific types of *subset effects*. GEO DataSets are partitioned into subsets that reflect experimental design. Profiles are flagged as having subset effects if they display differential expression across experimental variables. The subset effect scoring method is *ad hoc*, taking into account group medians, means, deviations inside the groups, penalties, and arbitrary cutoff thresholds. This flag is simply an attempt to give potentially differentially regulated genes higher visibility, and is not intended to provide an absolute determination of significance. Three search terms were used with the following relevant results:

#### Search: (C9orf72) and “rank subset effect” (flag type)

The highlight of this search term was an analysis of skeletal muscles from patients with limb girdle muscular dystrophy 2A (LGMD2A). LGMD2A is a recessive genetic disorder caused by mutations in calpain 3 (*CAPN3*). This study showed that *C9ORF72* was downregulated in muscle samples from 10 patients with calpainopathy compared to muscle samples from 10 healthy controls.

#### Search: (C9orf72) and “value subset effect” (flag type)

Six studies unrelated to brain or the CNS were flagged by this search term.

#### Search: C9orf72 (gene symbol) and “sample outlier” (flag type)

Studies showing differential expression of *C9ORF72* of several brain-related disorders or processes were identified by this search term. Expression was decreased in amniotic fluid samples from pregnant women carrying fetuses with neural tube defects (NTDs) diagnosed during ultrasound examination and increased in immature dendritic cells subjected to hypoxia *in vitro*. Another study demonstrated that *C9ORF72* is overexpressed in cerebellar cortical samples from schizophrenia patients.

We also examined *C9ORF72* expression using the Allen Human Brain Atlas for the Developing Human Brain datasets and found areas of its expression in the occipital and temporal lobes, and cerebellar cortex (Allen Brain Atlas Resources, Seattle, WA, USA: Allen Institute for Brain Sciences, 2009^2^). The gene was proportionally more highly expressed in the cerebellum, cingulate sulcus, and hypothalamus, compared to the caudate, and temporal pole. Analysis of curated information in the BrainSpan Atlas of the Developing Human Brain^3^ also showed that *C9ORF72* expression at age 12 weeks pre-natally is greatest in the dorsolateral prefrontal cortex, ventrolateral prefrontal cortex, medial prefrontal cortex, and inferolateral temporal cortex. At age 6 months and between ages 1 and 3 years, expression decreases proportionally compared to the other stages in the aforementioned regions and in the orbital frontal cortex, mediodorsal nucleus of the thalamus, primary visual cortex, and hippocampus.

## Discussion

The highest prevalence of ALS is found in Finland and in a study of Finnish cases, over 40% of familial ALS was attributed to a locus on chromosome 9p21 (Laaksovirta et al., 2010). Cases of ALS, FTD, and FTD-ALS, with type II TAR DNA-binding protein-43 (TDP-43) pathology, have also been associated with chromosome 9. Three recent reports have identified a locus on chromosome 9p21 for familial autosomal dominant FTD-ALS (Mok et al., 2012; DeJesus-Hernandez et al., 2011; Laaksovirta et al., 2010). The repeat expansion of a non-coding GGGGCC hexanucleotide repeat in *C9ORF72* was associated with the disease in the large FTD-ALS kindred and TDP-43 based pathology. It has been proposed that the repeat expansion leads to the loss of one alternately spliced *C9ORF72* transcript and formation of nuclear RNA foci. The mutation is in a non-coding region of the gene with no known function. There may be 2–20 repeats of GGGGCC in the normal gene and several hundred more in the mutant versions. The expanded repeat is transcribed into RNA, causing the resultant protein to be misfolded, with toxic aggregates, disrupting cellular function. While the C9ORF72 gene is believed to be non-coding the RNA which it produces may interfere with expression of another gene or genes. Toxic attributes of the abnormal RNA has also been proposed (Renton et al., 2011). It has been suggested that there is a single founder for this form of the disease (Mok et al., 2012).

This repeat expansion was also found in one third of familial ALS cases of outbred European decent, and it was more than twice as common as mutations in the *SOD* gene as a cause of familial ALS, and more than three times as common as TDP-43, fused in sarcoma (FUS), or optineurin (OPTN) mutations combined. It has been proposed that the mutation accounts for ∼40% of cases of familial ALS in persons of European descent. *SOD1* mutations are thought to be responsible for ∼15% of familial ALS cases. Sporadic ALS may also be caused by the mutation. It was also noted that age of symptom onset varied widely in patients carrying the pathogenic hexanucleotide expansion, some not developing weakness until their ninth decade. The age of onset in our family is similar to that reported by others, and anticipation was not observed. The behavioral variant FTLD was also seen in the series of DeJesus-Hernandez et al. (2011), in most cases. In the Finnish cohort reported by Renton et al. (2011), several patients presented with non-fluent progressive aphasia.

We found no evidence for association of polymorphisms in *C9ORF72* with AD in a large GWAS dataset, as has been reported by Rollinson et al. (2012). Bioinformatic analysis of this gene using the GEO database showed changes in gene expression of *C9ORF 72* in patients with muscular dystrophy, NTDs, and schizophrenia. We also report analysis of gene expression in brain regions using the Allen Human Brain Atlas. Mutations in this recently reported gene are now believed to be the most common cause of inherited ALS and an important cause of inherited FTLD. Our work suggests that the gene may also be important in other neurological conditions.

These data, together with the recent previous reports, are consistent with the view that this repeat expansion underpins a large proportion of those families in which ALS and FTD co-occur. The mechanisms by which the repeat expansion causes dysfunction remains to be established.

## Conflict of Interest Statement

The authors declare that the research was conducted in the absence of any commercial or financial relationships that could be construed as a potential conflict of interest.

## Footnotes

^1^http://www.ncbi.nlm.nih.gov/geoprofiles

^2^http://www.brainmap.org

^3^http://alleninstitute.org/science/public_resources/brainspan.html
